# Epstein–Barr virus-associated smooth muscle tumor of the lung in a child: a case report and literature review

**DOI:** 10.3389/fmed.2026.1763172

**Published:** 2026-05-01

**Authors:** Xuelian Chang, Qian Li, Changxiao Li, Shuaishuai Liu, Yuexia Bai, Zhongxiao Zhang, Jing Ma

**Affiliations:** 1 Respriatory and Interventional Radiology, Children’s Hospital Affiliated to Shandong University/Jinan Children’s Hospital, Jinan, China; 2 Department of Pathology, Children’s Hospital Affiliated to Shandong University/Jinan Children’s Hospital, Jinan, China

**Keywords:** case report, child, Epstein–Barr virus-associated smooth muscle tumor, immune dysfunction, pulmonary neoplasm

## Abstract

**Background:**

Pulmonary Epstein–Barr virus-associated smooth muscle tumor (EBV-SMT) is extremely rare in children with congenital immunodeficiency. This case report describes the clinical details of a pediatric pulmonary EBV-SMT case.

**Case presentation:**

A 10-year-old boy presented with a three-year history of persistent cough and intermittent fever. Chest computed tomography (CT) revealed multiple nodules in both lungs. Histopathological examination of bronchoscopic excision specimens revealed spindle cell proliferation with a preliminary diagnosis of myofibromatosis, followed by immunohistochemical analysis showing positivity for smooth muscle actin (SMA), Ki-67 (5–8%), and calponin, while *in situ* hybridization showed diffuse positivity for Epstein–Barr virus-encoded RNA (EBER), confirming the diagnosis of EBV-SMT. Further immunological evaluation revealed suspected underlying immune dysfunction, characterized by persistently low CD4 + T-cell counts. The patient underwent multiple bronchoscopic interventions, including electrosnaring and forceps excision, as well as laser therapy, high-frequency electrocautery, and cryotherapy, to manage the largest pulmonary and intrabronchial lesions. He was also managed with regular intravenous immunoglobulin (IVIG) therapy and close clinical monitoring.

**Conclusion:**

Pulmonary EBV-SMT should be considered in pediatric patients with unexplained pulmonary nodules and underlying immunodeficiency. Early diagnosis is essential, and a combination of local tumor control with surgical or bronchoscopic interventions and immunomodulatory therapy may be necessary. However, these treatments may not prevent tumor recurrence and progression, highlighting the challenges in long-term management.

## Introduction

Epstein–Barr virus-associated smooth muscle tumor (EBV-SMT) is a rare neoplasm that typically arises in immunocompromised individuals, including those with congenital immunodeficiency, acquired immunodeficiency syndrome (AIDS), or recipients of solid organ or hematopoietic stem cell transplants (1). Pulmonary involvement in children is particularly uncommon. In the lungs, EBV-SMTs may remain asymptomatic or present with nonspecific respiratory symptoms due to tumor mass effects. The pathogenesis of this tumor remains unclear, but is speculated to be related to latent EBV infection in smooth muscle cells (2).

While surgical resection remains the primary treatment modality, adjunctive approaches, such as antiviral therapy, antiretroviral therapy, chemotherapy, and reduction of immunosuppressive agents, have been explored with variable success (3). Nevertheless, the overall prognosis remains poor, and mortality is often attributed to severe opportunistic infections rather than tumor burden. This underscores the urgent need for more effective therapeutic strategies and long-term management approaches.

Here, we report a rare pediatric case of pulmonary EBV-SMT in a patient with suspected underlying immune dysfunction, aiming to contribute to the limited clinical literature on its presentation, diagnosis, and therapeutic approach.

## Case presentation

A 10-year-old boy, was admitted to the Department of Respiratory Intervention, Children’s Hospital of Shandong University, on August 17, 2023, due to “a cough persisting for more than three years, a bronchial mass for two years, and a diagnosis of primary immunodeficiency for over one year.”

Since February 2020, the patient had experienced recurrent paroxysmal coughing, with yellow sputum and intermittent fever (up to 38.5 °C), without clear triggers. Despite multiple courses of antibiotics, symptoms persisted. The patient had no significant past medical history. His parents and 17-year-old sister were healthy, with no known genetic disorders.

In October 2020, bronchoscopy revealed a mass in the left main bronchus, initially suspected to be a granuloma. Two whole-exome sequencing tests for genetic disorders conducted during this period failed to yield a definitive diagnosis. The first genetic analysis did not identify any clinically definitive pathogenic variant closely related to the patient’s phenotype, although several variants of uncertain significance were reported. A subsequent whole-exome sequencing analysis performed in May 2022, including partial intronic regions, identified a heterozygous NFKB2 c.1459-1G > C splice-site variant that was considered potentially relevant to the clinical phenotype, although its pathogenic significance remained inconclusive. The patient was hospitalized repeatedly due to recurrent respiratory infections.

In May 2022, based on findings of granulomatous hyperplasia on bronchoscopy, reanalysis of sequencing data, and a history of recurrent infections with reduced serum immunoglobulin M (IgM) and persistently low CD4 + T-cell counts, the patient was clinically considered to have possible underlying immune dysfunction and was managed as suspected primary immunodeficiency. Regular monthly intravenous immunoglobulin G (IVIG) supplementation was initiated, despite IgG levels being within the normal to high-normal range during the initial years of monitoring. Nevertheless, symptoms worsened, with increasing cough, sputum production, and occasional dyspnea and wheezing. Follow-up bronchoscopy revealed a significant increase in the size of the left main bronchial mass, causing severe airway obstruction.

**Figure 1:**
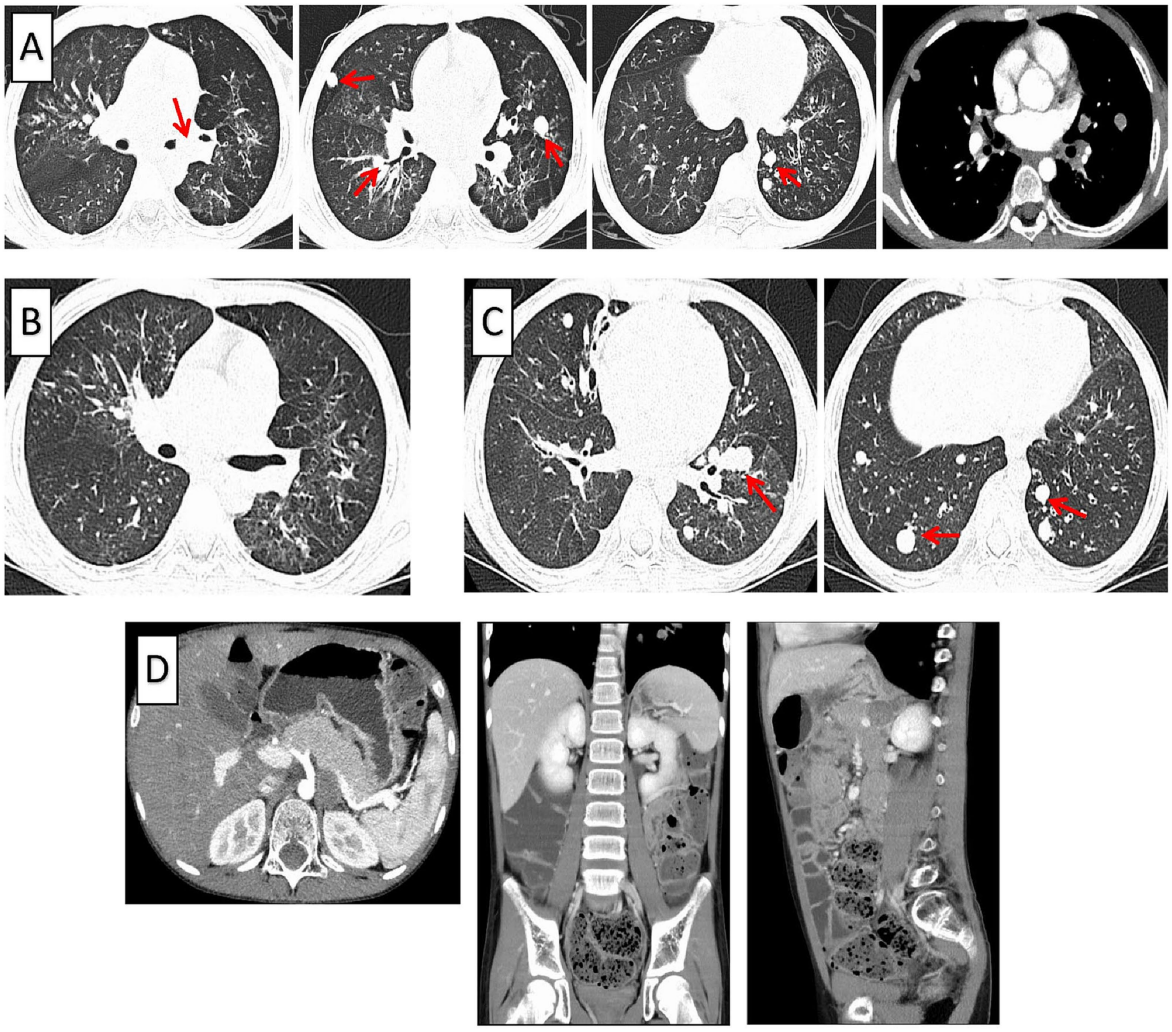
Chest computed tomography (CT) findings over time. (**A**) Chest CT on August 21, 2023, showing multiple nodular shadows distributed in both lungs and along some bronchi, with localized bronchiectasis in the left lower lobe. (**B**) Chest CT on September 13, 2023, demonstrating improved aeration of the left main bronchus following bronchoscopic resection of the endobronchial mass. (**C**) Chest CT on August 13, 2024, revealing an increased number and size of multiple round soft-tissue. Density nodules in the lower lobes of both lungs and bronchial involvement compared with prior imaging. (**D**) Abdominal plain and contrast-enhanced CT on August 15, 2024, depicting a lobulated, iso- to slightly hypodense mass in the left retroperitoneal adrenal region (approximately 2.6 cm× 1.4 cm× 2.6 cm) with poorly defined margins, an apparent capsule, and moderate enhancement with prominent capsular enhancement, indistinguishable from the left adrenal gland. Concentric soft-tissue. Density masses are also noted within the small intestine adjacent to the mid and lower poles of the left kidney, inseparable from the intestinal wall, with delayed enhancement.

On admission, physical examination showed mild respiratory distress, with oxygen saturation between 92 and 96% on room air. The patient appeared malnourished, and had generalized light brown skin pigmentation. A weakly positive three-concave sign was noted, and breath sounds were reduced on the left side. Laboratory testing revealed the presence of *Pseudomonas aeruginosa* in the metagenomic sequencing of bronchoalveolar lavage fluid (Table 1). Immunological evaluation demonstrated reduced immunoglobulin M levels, decreased total T lymphocytes, and reduced CD4 + helper/inducer T cells (Table 2). Pulmonary ultrasonography indicated localized pulmonary consolidation in the left lung. Chest computed tomography (CT) revealed multiple nodules within both lungs and parts of the bronchial tree, mediastinal lymphadenopathy, pneumonia, and mucus plug formation (Figure 1A).

**Table 1:** Relevant diagnostic indicators after admission.

Indicator	Measured value	Reference range
Complete Blood Count		
White Blood Cell Count	6.0	4.30~11.30 × 10 9/L
Red Blood Cell Count	5.26	4.2~5.7 × 10 12/L
Hemoglobin	120	118~156 g/L
Platelet Count	457	167 ~ 453 × 10 9/L
Neutrophil Count	3.23	1.6~7.8 × 10 9/L
Neutrophil Percentage	53.9%	31–70%
Lymphocyte Count	1.54	1.5~4.6
Lymphocyte Percentage	25.7%	23–59%
CRP	<0.499	<10 mg/L
Erythrocyte Sedimentation Rate (ESR)	3	0-15 mm/h
Epstein–Barr Virus (EBV) Antibody Testing		
EBV Viral Capsid Antigen IgM (EBV-VCA IgM)	<10.0 U/mL	Negative:<20/Indeterminate:20-40/Positive:>40.1
EBV Viral Capsid Antigen IgG (EBV-VCA IgG)	<141 U/mL	Negative:<20/Positive:>20.1
EBV Early Antigen IgG (EBV-EA IgG)	<5.00 U/mL	Negative:<10/Indeterminate:10-40/Positive:>40.1
EBV Nuclear Antigen IgG (EBV-NA IgG)	<326 U/mL	Negative:<5/Indeterminate:5-20/Positive:>20.1
Quantitative Measurement of EBV DNA	<103copy/ml	<103copy/ml

**Table 2:** Immunoglobulin and T-lymphocyte count results after disease onset.

Immunoglobulin panel (external laboratory)	Immunoglobulin G (6-13 g/L)	Immunoglobulin A (0.51–2.59 g/L)	Immunoglobulin M (0.4–1.8 g/L)	Complement C3 (0.89–1.87 g/L)	Complement C4 (0.14–0.44 g/L)
2020-10-03	7.65	0.55	0.27	1.52	0.44
2020-12-04	11.24	0.56	0.25	1.02	0.32
2021-03-05	12.6	0.71	0.3	1.05	0.31
2022-04-27	14.62	0.66	0.30	0.91	0.30
2023-01-24	11.69	0.61	0.32	1.51	0.53
2023-05-09	12.66	0.76	0.32	1.44	0.43

Immunoglobulin panel (Conducted at Our Institution)	Immunoglobulin G (0.7–15.3 g/L)	Immunoglobulin A (0.52–2.74 g/L)	Immunoglobulin M (0.29–1.41 g/L)	Complement C3 (0.83–1.61 g/L)	Complement C4 (0.13–0.41 g/L)
2023-08-18	12.3	0.595	0.170	1.05	0.29
2023-10-02	16.5	0.687	0.171		
2024-08-13	5.68	0.319	0.146		
2024-09-30	9.04	0.317	0.187		

TBNK	Total T Lymphocytes (55–82%)	CD4 (27–57%)	CD8 (14–34%)	NK (7.3–18.2%)	CD4/CD8 (1.1–1.4)
2020-10-03	52.78	10.97	27.78	26.03	0.46
2020-12-04	56.2	11.7	26.78	24.83	0.44
2023-05-10	49.68	15.4	23.76	28	0.65
2023-08-18	48.66	16.45	19.6	20.72	0.84

**Figure 2:**
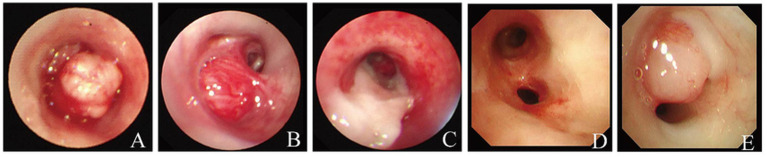
Bronchoscopic series of a left bronchial neoplasm: initial presentation, treatment response, mucosal repair, and recurrence **(A)** Bronchoscopic examination on August 23, 2023, revealing a neoplasm in the left main bronchus. **(B)** Neoplasm observed in the posterior basal segment bronchus of the left lower lobe. **(C)** Post-treatment appearance of the left main bronchus. **(D)** Bronchial mucosal repair observed in the left main bronchus after treatment on September 30, 2024. **(E)** Newly developed neoplasm in the apicoposterior segment of the left upper lobe.

**Figure 3:**
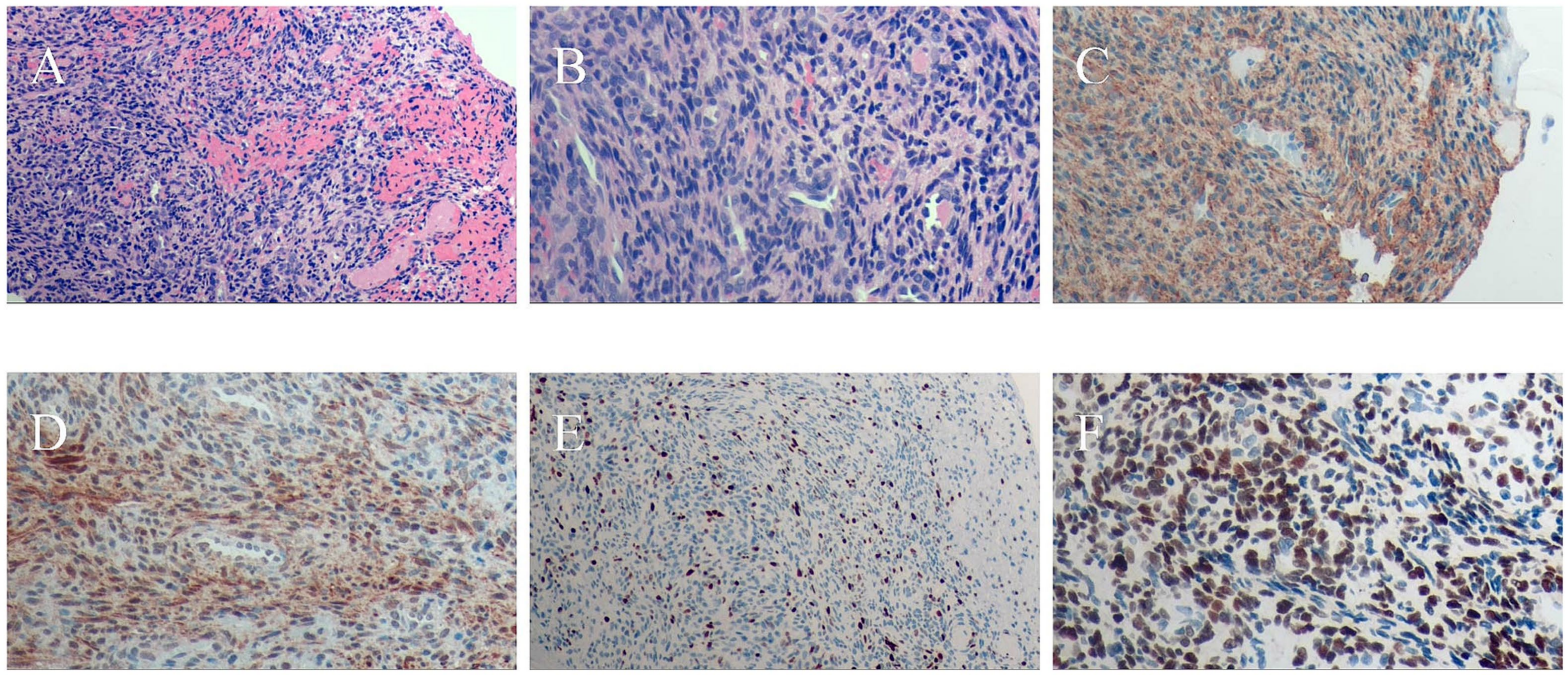
Histopathological examination of lung (bronchial) lesion biopsy. **(A)** Tumor cell invasion in the lung (bronchial) tissue (H&E staining, ×100). **(B)** Tumor cells exhibiting round, oval, and spindle shapes; most tumor cells were densely arranged, while some areas showed sparse cellularity with abundant vasculature. A perivascular pattern resembling pericytoma was observed in certain areas (H&E staining, ×200). **(C)** Immunohistochemistry showing diffuse cytoplasmic expression of SMA in tumor cells (IHC, ×200). **(D)** Immunohistochemistry showing diffuse cytoplasmic expression of Calponin in tumor cells (IHC, ×200). **(E)** Ki-67 proliferation index ranging from 5 to 8% (IHC, ×200). **(F)** *In situ* hybridization showing diffuse positivity for EBV-encoded RNA (EBER, ×200).

On August 23, 2023, the patient underwent bronchoscopic electrosnaring and forceps excision of the neoplasm in the left main bronchus. The initial pathological findings suggested myofibromatosis, with the possibility of mesenchymal tumor associated with genetic alterations not excluded (Figures 1B, 2A–C). Immunohistochemical analysis showed positivity for Vimentin, smooth muscle actin (SMA), Ki-67 (5–8%), and Calponin. *In situ* hybridization was positive for Epstein–Barr virus-encoded RNA (EBER) (Figure 3). The final diagnosis of EBV-SMT was established.

During hospitalization, the patient received anti-infective therapies, including cefoperazone-sulbactam, voriconazole, and linezolid. Multiple subsequent bronchoscopic procedures involving laser therapy, high-frequency electrocautery, electrosnaring, and cryotherapy were performed to excise or ablate recurrent intrabronchial neoplasms. The patient also continued to receive IVIG.

**Figure 4:**
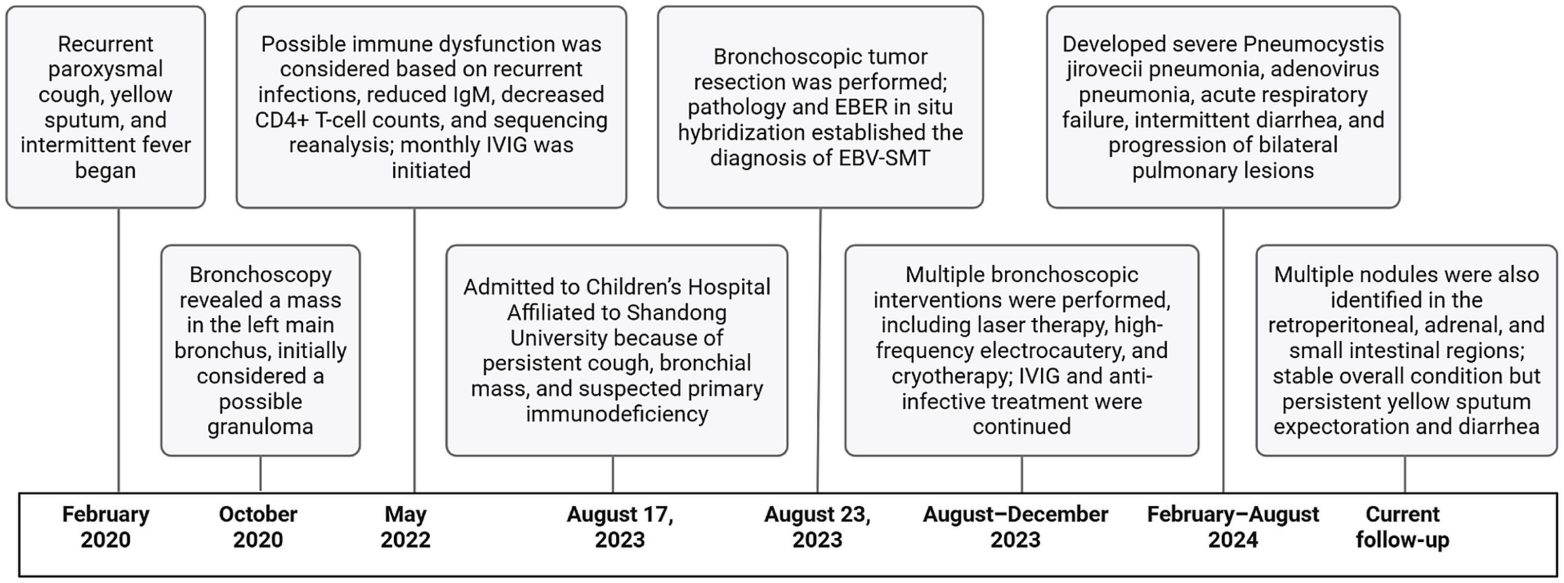
Timeline of the patient’s clinical course.

Despite initial improvement, the patient continued to experience recurrent respiratory tract infections. In February 2024, he was re-hospitalized due to severe Pneumocystis jirovecii pneumonia, adenovirus pneumonia, and acute respiratory failure. From May 2024, intermittent diarrhea developed. Chest CT in August 2024 revealed an increased number and size of multiple quasi-round soft tissue density lesions in both lungs (Figure 1C). Abdominal ultrasonography and CT suggested the presence of multiple nodules in the left retroperitoneum, adrenal region, and small intestine (Figure 1D). Colonoscopy was recommended but declined by the parents. The patient underwent additional bronchoscopic cryotherapy and remains under close follow-up. His overall condition is stable, though yellow sputum expectoration and diarrhea persist (Figures 2D,E). The major clinical events in the patient’s course are summarized in Figure 4.

## Discussion

This case report presents a rare instance of pulmonary EBV-SMT in a 10-year-old boy with suspected underlying immune dysfunction. Given the rarity of EBV-SMT in pediatric populations, particularly in the context of congenital immunodeficiency, this case may help increase awareness of the diagnostic challenges and potential management considerations associated with pulmonary EBV-SMT.

EBV-SMT was first reported in a transplant recipient in 1970 by Pritzker et al. (4), with the association between EBV infection and smooth muscle tumors established in 1995 by Lee et al. (5). Subsequent molecular studies have provided further insight into the role of EBV in the pathogenesis of these tumors. Jonigk et al. (6) demonstrated that EBV-associated smooth muscle tumors exhibit activation of signaling pathways including PI3K/AKT/mTOR, supporting a direct oncogenic role of EBV in smooth muscle cell proliferation. More recent clinicopathologic and molecular analyses have further shown that EBV-SMT represents a biologically distinct entity compared with conventional leiomyoma and leiomyosarcoma, characterized by EBV-driven transcriptional programs and distinct molecular features (7) (Table 3).

**Table 3:** Patient information for pulmonary EBV-associated smooth muscle tumors (literature review and current cases).

Case no.	Authors, year	Age at diagnosis/sex	Type of tumor	Tumor site	Single/multiple tumor nodules	Clinical feature	EBV status	Underlying immunodeficient state	Treatment	Outcome
1	Lee et al., 1995 (5)	6 yr./F	SMT	Lung, liver, retroperitoneum, stomach, colon	MN		EBV+, EBER+	Liver transplant	RI, AV, CT	Dead with disseminated candidal infection
2	Timmons et al., 1995 (26)	9 yr./M	High-gradeleiomyosarcoma	Liver, lung	MN		EBER+	Liver transplant	CT, RI	died of disease
3	Bluhm et al., 1997 (27)	35 yr./M	SMT	Endobronchial	MN		EBER+	AIDS	SR	Alive with disease
4	Jenson et al., 1997 (28)	8 yr./F	Leiomyosarcoma	Lung	N/A		EBV+, EBER+	AIDS	Unknown	Unknown
5	Jenson et al., 1997 (28)	4 yr./M	Leiomyoma	Lung	N/A		EBV+, EBER+	AIDS	Unknown	Unknown
6	Somers et al., 1998 (29)	15 yr./M	Leiomyosarcoma	Lung, allograft	MN		EBV+	Heart/Lung transplant	RI, AV	Dead with bronchopneumonia
7	Uribe-Uribe et al., 1998 (30)	28 yr./M	Leiomyosarcoma	Liver, lung, spleen, lymph nodes, thigh	MN		EBV+	kidney transplant	Unknown	Dead
8	Tulbah et al., 1999 (31)	6 yr./M	Leiomyosarcoma	Thyroid, liver, lung	MN		EBER+	Congenital T cell immunodeficiency	Unknown	Lost to follow up
9	To et al., 2000 (32)	38 yr./M	SMT	Bone, liver, lung, spleen	MN		EBV+	kidney transplant	RI, CT	Alive with disease
10	Collins et al., 2001 (33)	7 yr./F	SMT	Lntracranial, endobronchial	MN		EBV+, EBER+	Heart transplant	RI, SR	Alive, stable disease 2 yr. post-second resection
11	Monforte-Muñoz et al., 2003 (34)	8 yr./F	Leiomyomatosis	Gall bladder, liver, spleen,pancreas, intestinal tract, lung	MN		EBV+, EBER+	SCID	Unknown	Dead
12	Ferri et al., 2003 (35)	61 yr./F	Leiomyosarcoma	Lung	SN		EBV+, EBER+	Kidney transplant	SR	Alive and cured
13	Boudjemaa et al., 2004 (36)	5 yr./M	SMT	Brain, lung, liver, spleen, lymph node	MN		EBV+, EBER+	Kidney transplant	RI, CT	Deceased, brain tumor, seizure 2 months
14	Suankratay et al., 2005 (37)	38 yr./M	SMT	Spinal cord, gallbladder, lung, liver	MN		EBV+, EBER+	HIV positive	ART	Dead with disease
15	Hatano et al., 2006 (38)	6 yr./M	Leiomyoma	Lung	SN			Cellular and complement immunodeficiency	SR	Tumor-free for >2 years
16	Deyrup et al., 2006 (39)	50 yr./F	SMT	Glottic, lung, extra dural	N/A		EBV+, EBER+	Renal transplant	SR	Dead with disease
17	Deyrup et al., 2006 (39)	29 yr./M	SMT	Lung	N/A		EBV+, EBER+	Kidney transplant	Unknown	Alive with disease
18	Deyrup et al., 2006 (39)	52 yr./M	SMT	Lung	N/A		EBV+, EBER+	Kidney transplant	Unknown	Alive with disease
19	Atluri et al., 2007 (40)	6 yr./M	Leiomyomatosis	Lung, bilateral renal	MN		Negative	SCID (IL2RG)	Donor lymphocyte infusion	Tumor stable for >2 years
20	Nur et al., 2007 (41)	24 yr./M	Leiomyosarcoma	Lungs, liver, small bowel, retroperitoneum	MN		EBER+	Heart transplant	Unknown	Dead
21	Sprangers et al., 2008 (22)	51 yr./F	SMT	Lung, chest wall	MN		EBV+	kidney transplant	RI, AV	Alive with disease
22	Ong et al., 2009 (42)	31 yr./M	SMT	Glottic, pharynx, lungs, adrenal, liver, spleen, kidney	MN		EBER+	Renal transplant	Unknown	Alive with disease
23	Ong et al., 2009 (42)	52 yr./F	SMT	Glottic, lung, liver, spine	MN		EBER+	Renal transplant	SR	Dead with disease
24	Metta et al., 2009 (43)	36 yr./M	leiomyoma	Endobronchial	SN		EBER+	AIDS	SR	Alive with disease
25	Al Hussain et al., 2010 (44)	33 yr./M	Leiomyoma	Liver, mesentery, lung	MN		EBER+	Kidney transplant	SR, AV	Alive with disease
26	Suzuki et al., 2011 (45)	8 yr./F	SMT	Lung	MN	Repeated bronchitis	EBER+	Kidney transplant	SR, RI, AV	Alive, disease free 2 yr
27	Jonigk et al., 2012 (6)	7 yr./F	SMT	Bronchus	MN		EBER+	Liver transplant	-	-
28	Alabdulqader et al., 2012 (46)	44 yr./F	SMT	Liver, lung	SN		EBER+	renal transplant	SR	Dead
29	Petersson et al., 2013 (47)	37 yr./M	SMT	Intracranial, endobronchial, paraspinal	MN		EBER+	AIDS	SR	-
30	Jericho et al., 2014 (48)	2.5 yr./F	leiomyosarcoma	Liver, lung, spleen	MN		EBER+	Liver transplant	RI, SR/rituximab	Alive, stable disease at 2 yr
31	Dominelli et al., 2014 (49)	38 yr./F	SMT	Tonsil, lung, trachea	MN		EBV-	HIV positive	ART、SR	Alive with disease
32	Kazmi et al., 2014 (50)	8 yr./F	SMT	Adrenal, small bowel, lung, brain	MN		EBER+	Post-renal transplant	RI, SR, AV	Alive with disease
33	Adam et al., 2014 (51)	20 yr./F	SMT	Lung, left mainstem bron-Chus, adrenal	MN		EBV+, EBER+	HIV positive	HAART, SR	Alive with disease
34	Brescia et al., 2015 (52)	71 yr./M	SMT	Lung	SN		EBV+, EBER+	lung transplant	SR	Alive with disease
35	Arva et al., 2016 (53)	19 yr./F	SMT	Orbital, lung, large bowel PTLD	SN		EBV-, EBER+	heart transplant	partial resection	Alive with disease
36	Schober et al., 2017 (54)	7 yr./F	SMT	Gut, liver, lung, spleen, kidney, brain	MN		EBER+	CARMIL2 deficiency	SR, CT	Died of EBV + SMT-induced multi-organ Failure / Progressive disease at 12 years of age
37	Schober et al., 2017 (54)	8 yr./M	SMT	Gastrointestinal, pulmonary, liver	N/A		EBER+	CARMIL2 deficiency	CT	Died at 14 yr. of age
38	Huang Hui et al., 2017 (47)	6 yr./M	SMT	Endobronchial	SN		EBER+	Suspected low cellular immune function	SR	Alive with disease
39	Vaideeswar al., 2019 (55)	31 yr./M	leiomyomas	Lung	SN		EBER+	kidney transplant	Unknown	Dead
40	Yonkof et al., 2020 (56)	11 yr./M	SMT	Brain, para-spinal, lungs, colon, gallbladder, kidneys, liver	MN		EBER+	CARMIL2 deficiency	SR, CT, stem cell transplantation	Died at day 32 after peripheral blood stem cell transplantation
41	Yonkof et al., 2020 (56)	9 yr./M	SMT	Brain, ungs, liver, spleen	MN		EBER+	CARMIL2 deficiency	Not available	Alive with disease
42	Hansen et al., 2021 (57)	20 yr./F	Leiomyoma	Lungs, liver, colosigmoid junction, spleen	MN		EBV+, EBER+	Cardiac transplantation	CT	Alive with disease
43	Singh et al., 2021 (58)	39 yr./F	SMT	Endobronchial, adrenal	SN		EBER+	kidney transplant	RI, SR	Alive with disease
44	Shi et al., 2021 (59)	7 yr./F	SMT	Lumbar, cerebral, lung	MN		EBV+, EBER+	deletion mutation in the ITK gene	SR	Alive with disease
45	Khan et al., 2022 (1)	20 yr./M	Leiomyoma	Lung, thymus, liver, mesenteric, and retroperitoneal lymph nodes, ascending colon, and proximal left femoral bone marrow	MN		EBV˗, EBER+	heart transplant	Supportive	Dead
46	Afshin et al., 2022 (1)	16 yr./F	Leiomyoma	Lung, brain, kidney	MN		EBV˗, EBER+	heart transplant	CT、T-cell therapy	Alive with disease
47	Afshin et al., 2022 (1)	61 yr./M	Leiomyoma	Lung, kidney, liver,	MN		EBV+, EBER+	heart and liver transplant	RI、SR	Dead
48	Tang et al., 2022 (60)	7 yr./M	SMT	Left principal bronchus	SN		EBER+	Unknown	SR、CT	tumour progression
49	Wen et al., 2023 (61)	6 yr./F	Unknown	Lung, near the intracranial cavernous sinus, in the lumbar spine and paraspinal soft tissues	MN		EBV+, EBER+	IL-2 inducible T-cell kinase mutation	SR、CT	Alive with disease
50	Miyahara et al., 2023 (62)	20 yr./F	Leiomyoma	Lung, liver	MN		EBER+	kidney transplant	Everolimus; Stop immunosuppressant	Alive with disease
51	Sharma et al., 2023 (63)	34 yr./M	Leiomyoma	Lung	MN		EBER+	HIV positive	ART, SR	Alive with disease
52	Fu et al., 2024 (64)	32 yr./F	SMT	Lung	MN		EBV+, EBER+	kidney transplant	RI	Alive with disease
53	Fu et al., 2024 (64)	41 yr./F	SMT	Lung	MN		EBV˗, EBER+	kidney transplant	RI, SR	Alive with disease
54	Cobenas et al., 2025 (65)	14 yr./F	SMT	Spleen, lung, liver, central nervous system (CNS)	MN		EBV+, EBER+	kidney transplant	RI, SR	Alive with disease
55	Current case	10 yr./M	SMT	Lung	MN		EBV+, EBER+	Immune deficiency	The tumor was resected by bronchoscopy	Alive with disease

yr, year; F, female; M, male; SMT, smooth muscle tumor; MN, multiple nodules; SN, single nodule; EBER, Epstein–Barr virus-encoded small RNA; SCID, Severe combined immunodeficiency; RI, reduced immunosuppresion; CT, chemotherapy; SR, surgical resection; AV, antiviral therapy; HAART, Highly Active Anti-Retroviral Therapy; ART, Anti-Retroviral Therapy.

Although classically linked to transplantation or HIV, a few cases have been reported in individuals with no clearly defined immune deficiency. For example, Kang et al. (8) reported a six-year-old girl who developed EBV-SMT after immunosuppressive therapy for juvenile idiopathic arthritis, and Dantas Soares et al. (9) described a malnourished child with laryngeal EBV-SMT despite being HIV-negative and without transplant history. EBV-SMT is a distinctive mesenchymal neoplasm typically arising in individuals with impaired immune function, including those with HIV infection, post-transplant immunosuppression, or primary immunodeficiencies (10). To date, cases have been reported in both pediatric and adult populations, with no evident sex predilection, although a slight female predominance has been noted. 25 of the 54 reported patients (46.3%) were children aged 18 years or younger, underscoring the importance of considering EBV-SMT in pediatric populations with immune dysfunction.

Based on the etiology of immunodeficiency, EBV-SMT can be classified into three categories: congenital immunodeficiency-associated SMT, acquired immunodeficiency (HIV)-associated SMT, and post-transplantation immunosuppression-associated SMT (10). EBV-SMT has also been reported in association with specific inborn errors of immunity (IEI). Current IEI classification emphasizes that diagnosis should be based on integrated clinical, immunologic, and genetic evaluation rather than a single parameter such as CD4 lymphopenia alone (11). Among the reported IEI associated with EBV-SMT, CARMIL2 deficiency has been described in several pediatric cases, and GATA2 deficiency has also been reported in this setting (12). In our review of 54 previously reported patients, the underlying immune conditions included post-transplant immunosuppression in 33 cases (61.1%), congenital immunodeficiency in 8 cases (14.8%), HIV/AIDS in 9 cases (16.7%), and other or unclear causes in 4 cases (7.4%).

In our patient, recurrent infections, reduced IgM levels, and persistently decreased CD4 + T-cell counts suggested underlying immune dysfunction, although a definitive genetic diagnosis was not established. Two separate genetic evaluations did not identify a previously established pathogenic variant that could definitively explain the immune phenotype. Therefore, in this case, the diagnosis of immune dysfunction remained primarily based on the clinical history and immunologic findings rather than molecular confirmation alone.

Although EBV-SMT most commonly occurs in patients with congenital or acquired immunodeficiency, rare cases have been reported in individuals without clinically evident immune impairment. Reported examples include intracranial EBV-associated leiomyosarcoma described by Takei et al. (13), synchronous skull base and adrenal EBV-SMT reported by Brown et al. (14), and EBV-SMT of the cranio-cervical junction reported by Lau et al. (15) These reports indicate that, although uncommon, EBV-SMT can occasionally occur in patients without a clearly established immunodeficiency. Because a definitive genetic diagnosis of an inborn error of immunity was not established in our patient, the possibility that this tumor developed in the setting of undetected or subtle immune dysregulation, or less likely in an immunocompetent host, cannot be completely excluded.

Histologically, EBV-SMTs show fascicles of spindle-shaped cells with eosinophilic cytoplasm and positive immunoreactivity for smooth muscle markers (SMA, calponin, and desmin) and EBER (10, 16). Calponin is included as part of the routine smooth muscle immunohistochemical panel used in our institution and showed clear positivity in this case, supporting smooth muscle differentiation, although h-caldesmon is also widely used as a marker of smooth muscle differentiation in many studies. The tumor in our case displayed a hemangiopericytoma-like growth pattern, consistent with reports that describe variable morphology in EBV-SMTs (17). For instance, Lee et al. noted that some EBV-SMTs exhibit hemangiopericytoma-like vascular proliferation, underscoring the morphological diversity of this tumor type (17). Given the lack of histological specificity, EBER *in situ* hybridization remains critical for definitive diagnosis.

The differential diagnosis of EBV-SMT includes several spindle cell lesions that may occur in similar clinical settings. In immunocompromised patients, particularly those with HIV infection, Kaposi sarcoma should be considered because it can present as a spindle cell tumor involving visceral organs. In pediatric patients, inflammatory myofibroblastic tumor may arise in the lung and may show overlapping spindle cell morphology. In congenital pulmonary tumors, fetal lung interstitial tumor (FLIT) represents another rare spindle cell lesion that may enter the differential diagnosis in infants and young children. In addition, EBV-positive inflammatory follicular dendritic cell sarcoma has recently been described as an EBV-associated spindle cell neoplasm with a broad morphological spectrum and should also be distinguished using appropriate immunophenotypic markers (18, 19).

EBV-SMTs can arise in multiple anatomical sites within the same patient. Among the 54 previously reported patients reviewed, 38 (70.4%) had multifocal tumors, 10 (18.5%) had single-nodule lesions, and the remainder were unspecified, suggesting that multifocality is a hallmark of EBV-SMT regardless of the underlying immune context. In cases associated with congenital immunodeficiency, the most frequently affected sites include the lungs and larynx, followed by intracranial tumors, liver, adrenal glands, and spleen.

The clinical presentation of EBV-SMT varies by tumor location. In our patient, intrabronchial obstruction led to impaired pulmonary clearance and recurrent infections. Bronchoscopic interventions, including laser therapy and cryotherapy, temporarily relieved airway obstruction. However, persistent tumor regrowth and delayed mucosal healing indicated limitations of surgical treatment alone.

Unlike typical endobronchial tumors in immunocompetent children, healing in this patient was impaired by underlying immune dysfunction. At one-year follow-up, the patient developed intermittent abdominal pain and diarrhea. Further evaluation could not exclude the possibility of EBV-SMT involving the adrenal glands and small intestine. Among the 54 previously reported cases, 17 involved only the lungs and bronchi; seven involved both lungs and intestines; three involved lungs and adrenal glands; and one involved lungs, adrenal glands, and small intestine. These findings indicate that, in the setting of congenital immunodeficiency, EBV-SMT may present either as disease confined to the lung/bronchial tree or as involvement of multiple organs, consistent with our case.

Previous reports have also highlighted malnutrition as a potential contributing factor in the pathogenesis of EBV-SMT due to its impact on immune function (20). Our patient exhibited marked malnutrition, which may have exacerbated immune suppression and tumor susceptibility. However, in this patient, malnutrition developed after the onset of recurrent respiratory infections and was therefore considered more likely to be a contributing or aggravating factor rather than the primary driver of tumor development. Notably, in addition to the pulmonary lesions, follow-up imaging suggested the possibility of EBV-SMT involvement in the adrenal gland and small intestine. This aligns with earlier studies indicating that EBV-SMT can occur at multiple anatomical sites in the same patient, particularly in immunocompromised children (9).

The current management of EBV-SMT lacks standardization. Surgical resection remains the mainstay, particularly for symptomatic lesions (21). However, multifocality or deep anatomical location often precludes complete resection. In select cases, adjuvant approaches such as antiviral therapy and immunosuppressant modulation, including substitution of cyclosporine with sirolimus, have been associated with tumor regression in adult patients (22). Sirolimus, an mTOR pathway inhibitor, may suppress EBV-driven tumor growth, although pediatric data are limited. These observations are consistent with previous reports suggesting that EBV-SMT often demonstrates relatively indolent clinical behavior compared with conventional leiomyosarcoma, and that modulation of the patient’s immune status together with mTOR-targeted therapy may contribute to disease control.

Rituximab was not administered in our patient. Although rituximab has been reported in selected EBV-SMT cases, it was not used in this case because the child had recurrent respiratory tract infections and clinically suspected underlying immunodeficiency, while the genetic basis of the immune disorder remained inconclusive. Given the potential immunosuppressive effect of rituximab on B-cell function, the treating team considered that the risk of further infectious complications might outweigh its uncertain benefit at that stage. After submission of the manuscript, additional organoid-based drug screening was performed, which showed relatively low sensitivity to both rituximab and rapamycin. Based on these findings, anti-tumor drug therapy was added, and the child remains under follow-up.

In a case reported by Sprangers et al. (22), pulmonary EBV-SMT resolved completely following discontinuation of cyclosporine and initiation of ganciclovir. Sirolimus has also been proposed as a therapeutic alternative for its dual role as an immunosuppressant and anti-tumor agent via AKT–mTOR inhibition. Its use in EBV-SMT highlights the potential for targeted molecular therapy beyond surgical resection alone (23).

For HIV-associated SMT, antiretroviral therapy (ART) has demonstrated therapeutic benefits by restoring immune function and suppressing tumor progression (24). Across the reviewed cases, treatment strategies varied widely and included surgical resection, reduction of immunosuppression, antiviral therapy, chemotherapy, or combinations of these approaches, reflecting the absence of a standardized treatment strategy. Nevertheless, long-term outcomes remain poor. Among 25 reported pediatric cases of immunodeficiency-associated EBV-SMT, 8 resulted in death, primarily from infections rather than tumor burden (25). Our case similarly underscores that infection-related complications pose the greatest threat to survival.

This case report provides a detailed description of a pediatric case of EBV-associated pulmonary smooth muscle tumor. It highlights the need for individualized multimodal strategies that combine local tumor control, supportive care, and targeted systemic therapies. Clinicians should maintain a high index of suspicion for EBV-SMT in immunocompromised pediatric patients presenting with respiratory symptoms and intrapulmonary lesions. Nevertheless, this study has several limitations. As a single case report, it cannot establish causal relationships or generalizable treatment outcomes. Furthermore, the patient’s genetic basis of immunodeficiency remains unconfirmed, limiting mechanistic insight. Future research should include larger cohorts and integrate molecular analysis to guide personalized management of EBV-SMT.

## Conclusion

This case highlights the diagnostic and therapeutic challenges of pulmonary EBV-associated smooth muscle tumors in children with suspected underlying immune dysfunction. While surgical intervention provided temporary relief by alleviating airway obstruction, tumor regrowth and recurrent infections persisted, indicating that surgery alone is insufficient for long-term disease control. This case underscores the need for individualized treatment strategies that combine local and systemic approaches, together with ongoing immune monitoring and immunological evaluation to address both the tumor and the underlying immune dysfunction. Therapeutic decision-making should carefully balance potential anti-tumor benefit against the risk of exacerbating infection in children with suspected immune dysfunction. Furthermore, our case reinforces the observation that opportunistic infections, rather than tumor burden, often pose the greatest threat to survival in these patients. However, as a single case report, these observations must be interpreted with caution.

## Data Availability

The original contributions presented in the study are included in the article/supplementary material, further inquiries can be directed to the corresponding author.

## References

[1] Khan AA Estfan BN Yalamanchali A Niang D Savage EC Fulmer CG . Epstein-Barr virus-associated smooth muscle tumors in immunocompromised patients: six case reports. World J Clin Oncol. (2022) 13:540–52. doi: 10.5306/wjco.v13.i6.540, 35949429 PMC9244966

[2] Young SM Amrith S Ting E Wu B Nga ME Sundar G. "EBV smooth muscle tumour". In: Amrith S Sundar G Young SM, editors. Ocular Adnexal Lesions: A Clinical, Radiological and Pathological Correlation. Singapore: Springer Singapore (2019). p. 257–61.

[3] Chong YB Lu PL Ma YC Yin HL Chang CH. Epstein-Barr virus-associated smooth muscle tumor and its correlation with CD4 levels in a patient with HIV infection. Front Cell Infect Microbiol. (2022) 12:725342. doi: 10.3389/fcimb.2022.725342, 35141174 PMC8818939

[4] Pritzker KP Huang SN Marshall KG. Malignant tumours following immunosuppressive therapy. Can Med Assoc J. (1970) 103:1362–5. 4923839 PMC1930644

[5] Lee ES Locker J Nalesnik M Reyes J Jaffe R Alashari M . The association of Epstein-Barr virus with smooth-muscle tumors occurring after organ transplantation. N Engl J Med. (1995) 332:19–25. doi: 10.1056/NEJM199501053320104, 7990861

[6] Jonigk D Laenger F Maegel L Izykowski N Rische J Tiede C . Molecular and clinicopathological analysis of Epstein-Barr virus-associated posttransplant smooth muscle tumors. Am J Transplant. (2012) 12:1908–17. doi: 10.1111/j.1600-6143.2012.04011.x, 22420456

[7] Wah NW Mok Y Omar N Chang KTE Tay TKY Hue SS . Clinicopathologic and molecular characteristics of Epstein-Barr virus-associated smooth muscle tumor compared with those of leiomyoma and Leiomyosarcoma. Mod Pathol. (2023) 36:100127. doi: 10.1016/j.modpat.2023.100127 36965331

[8] Kang Z Xu J Li Z. Juvenile idiopathic arthritis with Epstein-Barr virus-associated smooth muscle tumor in a 6-year-old girl: a rare case report. Front Pediatr. (2021) 9:680113. doi: 10.3389/fped.2021.680113, 34222149 PMC8249757

[9] Jossen J Chu J Hotchkiss H Wistinghausen B Iyer K Magid M . Epstein-Barr virus-associated smooth muscle tumors in children following solid organ transplantation: a review. Pediatr Transplant. (2015) 19:235–43. doi: 10.1111/petr.12426 25572657

[10] Dekate J Chetty R. Epstein-Barr virus-associated smooth muscle tumor. Arch Pathol Lab Med. (2016) 140:718–22. doi: 10.5858/arpa.2015-0120-RS 27362573

[11] Tangye SG Al-Herz W Bousfiha A Cunningham-Rundles C Franco JL Holland SM . Human inborn errors of immunity: 2022 update on the classification from the International Union of Immunological Societies Expert Committee. J Clin Immunol. (2022) 42:1473–507. doi: 10.1007/s10875-022-01289-3, 35748970 PMC9244088

[12] Magg T Schober T Walz C Ley-Zaporozhan J Facchetti F Klein C . Epstein-Barr virus(+) smooth muscle tumors as manifestation of primary immunodeficiency disorders. Front Immunol. (2018) 9:368. doi: 10.3389/fimmu.2018.00368, 29535735 PMC5835094

[13] Takei H Powell S Rivera A. Concurrent occurrence of primary intracranial Epstein-Barr virus-associated leiomyosarcoma and Hodgkin lymphoma in a young adult. J Neurosurg. (2013) 119:499–503. doi: 10.3171/2013.3.JNS121707 23621602

[14] Brown DA Deep NL Driscoll CL Link MJ Jentoft ME Daniels DJ. Synchronous Epstein-Barr virus-associated skull base and adrenal smooth-muscle tumors in an 8-year-old girl with recent Epstein-Barr virus infection. J Neurosurg Pediatr. (2018) 22:283–7. doi: 10.3171/2018.3.PEDS17609, 29905497

[15] Lau KW Hsu YW Lin YT Yeap MC Lee CC Chen KT. Case history on Epstein-Barr virus-associated smooth muscle tumor (EBV-SMT) of cranio-cervical junction in an immunocompetent patient. Br J Neurosurg. (2021) 37:1815–9. doi: 10.1080/02688697.2021.1932745 34057864

[16] Bourhis A Roussel-Robert V Viard JP Peyre M Bielle F. A case of Epstein-Barr virus-associated smooth muscle tumor of the posterior interosseous nerve mimicking schwannoma. Neuropathology. (2022) 42:52–7. doi: 10.1111/neup.12774, 35026862

[17] Lee OZJ Omar N Tay JK Lee VKM. A Clinicopathology review and update of Epstein-Barr virus-associated mesenchymal tumors. Cancers. (2023) 15:15. doi: 10.3390/cancers15235563, 38067267 PMC10705784

[18] Chen S You Z Chen X Wang C. Clinicopathological and molecular genetic insights into EBV-positive inflammatory follicular dendritic cell sarcoma. Hum Pathol. (2024) 153:105668. doi: 10.1016/j.humpath.2024.105668, 39370049

[19] Li Y Yang X Tao L Zeng W Zuo M Li S . Challenges in the diagnosis of Epstein-Barr virus-positive inflammatory follicular dendritic cell sarcoma: extremely wide morphologic Spectrum and Immunophenotype. Am J Surg Pathol. (2023) 47:476–89. doi: 10.1097/PAS.0000000000002011 36574358

[20] Soares CD Carlos R Molina JPD de Lima Morais TM de Almeida OP. Laryngeal Epstein-Barr virus-associated smooth muscle tumor in an undernourished child. Head Neck Pathol. (2019) 13:722–6. doi: 10.1007/s12105-018-0960-0, 30120720 PMC6854134

[21] Lau KW Hsu YW Lin YT Chen KT. Role of surgery in treating epstein-barr virus-associated smooth muscle tumor (EBV-SMT) with central nervous system invasion: a systemic review from 1997 to 2019. Cancer Med. (2021) 10:1473–84. doi: 10.1002/cam4.3770, 33576167 PMC7940242

[22] Sprangers B Smets S Sagaert X Wozniak A Wollants E Van Ranst M . Posttransplant Epstein-Barr virus-associated myogenic tumors: case report and review of the literature. Am J Transplant. (2008) 8:253–8. doi: 10.1111/j.1600-6143.2007.02054.x, 18184312

[23] Hernando E Charytonowicz E Dudas ME Menendez S Matushansky I Mills J . The AKT-mTOR pathway plays a critical role in the development of leiomyosarcomas. Nat Med. (2007) 13:748–53. doi: 10.1038/nm1560, 17496901

[24] Ara T Endo T Goto H Kasahara K Hasegawa Y Yokoyama S . Antiretroviral therapy achieved metabolic complete remission of hepatic AIDS related Epstein-Barr virus-associated smooth muscle tumor. Antivir Ther. (2022) 27:135965352211268. doi: 10.1177/13596535221126828, 36112852

[25] Vij M Sivasankaran M Jayaraman D Sankaranarayanan S Kumar V Munirathnam D . CARMIL2 immunodeficiency with Epstein Barr virus associated smooth muscle tumor (EBV-SMT). Report of a case with comprehensive review of literature. Fetal Pediatr Pathol. (2022) 41:1023–34. doi: 10.1080/15513815.2021.2000533, 34738861

[26] Timmons CF Dawson DB Richards CS Andrews WS Katz JA. Epstein-Barr virus-associated leiomyosarcomas in liver transplantation recipients. Origin from either donor or recipient tissue. Cancer. (1995) 76:1481–9. doi: 10.1002/1097-0142(19951015)76:8<1481::aid-cncr2820760828>3.0.co;2-k 8620427

[27] Bluhm JM Yi ES Diaz G Colby TV Colt HG. Multicentric endobronchial smooth muscle tumors associated with the Epstein-Barr virus in an adult patient with the acquired immunodeficiency syndrome: a case report. Cancer. (1997) 80:1910–3. doi: 10.1002/(SICI)1097-0142(19971115)80:10<1910::AID-CNCR6>3.0.CO;2-R, 9366292

[28] Jenson HB Leach CT McClain KL Joshi VV Pollock BH Parmley RT . Benign and malignant smooth muscle tumors containing Epstein-Barr virus in children with AIDS. Leuk Lymphoma. (1997) 27:303–14. doi: 10.3109/10428199709059684, 9402327

[29] Somers GR Tesoriero AA Hartland E Robertson CF Robinson PJ Venter DJ . Multiple leiomyosarcomas of both donor and recipient origin arising in a heart-lung transplant patient. Am J Surg Pathol. (1998) 22:1423–8. doi: 10.1097/00000478-199811000-00014 9808136

[30] Uribe-Uribe NO Aviles-Salas A Orozco-Estevez H Alberu J Angeles-Angeles A. Leiomyosarcoma associated with Epstein-Barr virus in an adult with renal transplant. Rev Investig Clin. (1998) 50:255–8. 9763893

[31] Tulbah A Al-Dayel F Fawaz I Rosai J. Epstein-Barr virus-associated leiomyosarcoma of the thyroid in a child with congenital immunodeficiency: a case report. Am J Surg Pathol. (1999) 23:473–6. doi: 10.1097/00000478-199904000-00013, 10199478

[32] To K Lai FM Wang AY Leung CB Choi PC Szeto CC . Posttransplant Epstein-Barr virus-associated myogenic tumors involving bone. Cancer. (2000) 89:467–72. doi: 10.1002/1097-0142(20000715)89:2<467::AID-CNCR36>3.3.CO;2-3, 10918181

[33] Collins MH Montone KT Leahey AM Hodinka RL Salhany KE Clark BJ . Metachronous Epstein-Barr virus-related smooth muscle tumors in a child after heart transplantation: case report and review of the literature. J Pediatr Surg. (2001) 36:1452–5. doi: 10.1053/jpsu.2001.26396, 11528626

[34] Monforte-Munoz H Kapoor N Saavedra JA. Epstein-Barr virus-associated leiomyomatosis and posttransplant lymphoproliferative disorder in a child with severe combined immunodeficiency: case report and review of the literature. Pediatr Dev Pathol. (2003) 6:449–57. doi: 10.1007/s10024-003-8096-x, 14708738

[35] Ferri L Fraser R Gaboury L Mulder D. Epstein-Barr virus-associated pulmonary leiomyosarcoma arising twenty-nine years after renal transplantation. J Thorac Cardiovasc Surg. (2003) 126:877–9. doi: 10.1016/s0022-5223(03)00719-0, 14502177

[36] Boudjemaa S Boman F Guigonis V Boccon-Gibod L. Brain involvement in multicentric Epstein-Barr virus-associated smooth muscle tumours in a child after kidney transplantation. Virchows Arch. (2004) 444:387–91. doi: 10.1007/s00428-004-0975-7, 15143769

[37] Suankratay C Shuangshoti S Mutirangura A Prasanthai V Lerdlum S Shuangshoti S . Epstein-Barr virus infection-associated smooth-muscle tumors in patients with AIDS. Clin Infect Dis. (2005) 40:1521–8. doi: 10.1086/429830, 15844077

[38] Hatano M Takada H Nomura A Ohga S Ohshima K Saeki I . Epstein-Barr virus-associated bronchial leiomyoma in a boy with cellular immunodeficiency. Pediatr Pulmonol. (2006) 41:371–3. doi: 10.1002/ppul.20375, 16429426

[39] Deyrup AT Lee VK Hill CE Cheuk W Toh HC Kesavan S . Epstein-Barr virus-associated smooth muscle tumors are distinctive mesenchymal tumors reflecting multiple infection events: a clinicopathologic and molecular analysis of 29 tumors from 19 patients. Am J Surg Pathol. (2006) 30:75–82. doi: 10.1097/01.pas.0000178088.69394.7b, 16330945

[40] Atluri S Neville K Davis M Robertson KA Marshalleck FE O'Malley DP . Epstein-Barr-associated leiomyomatosis and T-cell chimerism after haploidentical bone marrow transplantation for severe combined immunodeficiency disease. J Pediatr Hematol Oncol. (2007) 29:166–72. doi: 10.1097/MPH.0b013e31803b95b3, 17356396

[41] Nur S Rosenblum WD Katta UD Islam H Brown K Ramaswamy G. Epstein-Barr virus-associated multifocal leiomyosarcomas arising in a cardiac transplant recipient: autopsy case report and review of the literature. J Heart Lung Transplant. (2007) 26:944–52. doi: 10.1016/j.healun.2007.05.022, 17845934

[42] Ong KW Teo M Lee V Ong D Lee A Tan CS . Expression of EBV latent antigens, mammalian target of rapamycin, and tumor suppression genes in EBV-positive smooth muscle tumors: clinical and therapeutic implications. Clin Cancer Res. (2009) 15:5350–8. doi: 10.1158/1078-0432.CCR-08-2979, 19706821

[43] Metta H Corti M Redini L Dure R Campitelli AM Narbaitz M. Endobronchial leiomyoma: an unusual non-defining neoplasm in a patient with AIDS. Rev Inst Med Trop Sao Paulo. (2009) 51:53–5. doi: 10.1590/s0036-46652009000100010 19229392

[44] Al Hussain T Haleem A Alsaad KO. Synchronous hepatic, mesenteric and pulmonary Epstein-Barr virus-associated smooth muscle tumors in a renal transplant recipient. Clin Transpl. (2010) 24:579–84. doi: 10.1111/j.1399-0012.2009.01206.x 20156224

[45] Suzuki K Urushihara N Fukumoto K Watanabe K Wada N Takaba E. A case of Epstein-Barr virus-associated pulmonary leiomyosarcoma arising five yr after a pediatric renal transplant. Pediatr Transplant. (2011) 15:E145–8. doi: 10.1111/j.1399-3046.2010.01329.x, 20456653

[46] Alabdulqader NA Yousef MM Bernacki KD Al-Abbadi MA. Epstein-Barr virus associated smooth muscle tumors. Synchronous liver and lung involvement. Saudi Med J. (2012) 33:1010–3. doi: 10.15537/1658-3175.5624 22964814

[47] Petersson F. Epstein-Barr virus-associated smooth muscle tumor-report of 3 tumors including 1 intracerebral case with a prominent intratumoral B-lymphocytic component and plasma cells. Ann Diagn Pathol. (2013) 17:91–8. doi: 10.1016/j.anndiagpath.2012.07.007, 23022018

[48] Jericho H Weinstein J Melin-Aldana H Leuer KC Wyers M Alonso EM . Successful treatment with rituximab of an Epstein-Barr virus-associated leiomyosarcoma occurring after liver transplantation. J Pediatr Gastroenterol Nutr. (2014) 58:e2–4. doi: 10.1097/MPG.0b013e31826f2786, 24378523

[49] Dominelli GS Jen R Park K Shaipanich T. Tracheal Epstein-Barr virus-associated smooth muscle tumour in an HIV-positive patient. Can Respir J. (2014) 21:334–6. doi: 10.1155/2014/984252, 25255459 PMC4266150

[50] Kazmi SA Aizenberg MR Harper JL McComb RD. Multifocal histologically malignant Epstein-Barr virus-associated smooth muscle tumor in a pediatric transplant patient with an indolent course. Int J Surg Pathol. (2014) 22:186–9. doi: 10.1177/1066896913494793, 23842005

[51] Adam E Wang L Herrington C Bliss D Church JA. Synchronous HIV/AIDS-related Epstein-Barr virus-associated smooth muscle tumors in a 20-year-old female. Pediatr Infect Dis J. (2014) 33:1055–6. doi: 10.1097/INF.0000000000000372 24759574

[52] Brescia AA Khullar OV Gal AA Neujahr D Force SD. Epstein-Barr virus-associated pulmonary smooth muscle tumor after lung transplantation. Ann Thorac Surg. (2015) 99:e145–6. doi: 10.1016/j.athoracsur.2015.02.097, 26046906

[53] Arva NC Schafernak KT. Rare presentations of Epstein-Barr virus--associated smooth muscle tumor in children. Pediatr Dev Pathol. (2016) 19:132–8. doi: 10.2350/15-05-1627-CR.1 26230054

[54] Schober T Magg T Laschinger M Rohlfs M Linhares ND Puchalka J . A human immunodeficiency syndrome caused by mutations in CARMIL2. Nat Commun. (2017) 8:14209. doi: 10.1038/ncomms14209, 28112205 PMC5473639

[55] Vaideeswar P Yadav S. Multifocal Epstein-Barr virus-associated miliary post-transplant smooth muscle tumors. Indian J Pathol Microbiol. (2019) 62:293–5. doi: 10.4103/IJPM.IJPM_485_18, 30971559

[56] Yonkof JR Gupta A Rueda CM Mangray S Prince BT Rangarajan HG . A novel pathogenic variant in CARMIL2 (RLTPR) causing CARMIL2 deficiency and EBV-associated smooth muscle tumors. Front Immunol. (2020) 11:884. doi: 10.3389/fimmu.2020.00884, 32625199 PMC7314954

[57] Hansen BT Bacher P Eiz-Vesper B Heckl SM Klapper W Koch K . Adoptive cell transfer of allogeneic Epstein-Barr virus-specific T lymphocytes for treatment of refractory EBV-associated Posttransplant smooth muscle tumors: a case report. Front Immunol. (2021) 12:727814. doi: 10.3389/fimmu.2021.727814, 34925312 PMC8677671

[58] Singh H Jani CT Abdalla M Puzyrenko A Kurman JS Benn BS. A 39-year-old woman with synchronous endobronchial and adrenal tumors. Chest. (2021) 160:e629–32. doi: 10.1016/j.chest.2021.07.028, 34872676

[59] Shi Q Tang WF He XL Tian X. Epstein-Barr virus-associated smooth muscle tumor in a girl. Zhongguo Dang Dai Er Ke Za Zhi. (2021) 23:739–42. doi: 10.7499/j.issn.1008-8830.2103152 34266534 PMC8292655

[60] Tang Jue LL Yihuan H Fenghua W Jianhua L Jiahang Z Dongmei H . Surgical treatment and outcome of primary tracheobronchial tumors in 15 children. *Chin*. J Thorac Cardiovasc Surg. (2022) 38:746–50. doi: 10.3760/cma.j.cn112434-20210407-00127

[61] Wen Q Ning J Mao Z Long X He X Chen Z . Case report: multiple epstein-barr virus-associated smooth muscle tumours in a child with IL-2-inducible T-cell kinase mutation of undetermined clinical significance. Front Pediatr. (2023) 11:1189219. doi: 10.3389/fped.2023.1189219, 37465420 PMC10350626

[62] Miyahara J Shimazu K Saito A Saito M Fukuda K Yoshida T . Clinical course of a rare Epstein-Barr virus-associated smooth muscle tumor and its genomic analysis. Case Rep Oncol. (2023) 16:577–84. doi: 10.1159/000530383, 37900829 PMC10601747

[63] Sharma S Ulicny J Thuzar M Aguiar RS Sharkey S Zhang F . Epstein-Barr virus-associated pulmonary leiomyoma in a patient with untreated human immunodeficiency virus infection. *Open forum*. Infect Dis. (2023) 10:ofad492. doi: 10.1093/ofid/ofad492, 37829442 PMC10566238

[64] Fu XY Gao X Zhao CL Qi XF Ouyang XJ Zhu LH . Pulmonary Epstein-Barr virus-associated smooth muscle tumor after kidney transplantation: two case reports with review of differential diagnosis. Romanian J Morphol Embryol. (2024) 65:107–12. doi: 10.47162/RJME.65.1.13, 38527990 PMC11146452

[65] Cobenas CJ Pereyra P Spizzirri AP Gauto Santacruz C Del Carmen SA Altamirano E . Epstein-Barr virus-associated post-transplant smooth muscle tumours in a kidney transplant patient. Pediatr Nephrol. (2025) 40:389–91. doi: 10.1007/s00467-024-06493-4, 39230732

